# Risk factors for prospective increase in psychological stress during COVID-19 lockdown in a representative sample of adolescents and their parents

**DOI:** 10.1192/bjo.2021.49

**Published:** 2021-05-03

**Authors:** Kerstin Paschke, Nicolas Arnaud, Maria Isabella Austermann, Rainer Thomasius

**Affiliations:** German Center for Addiction Research in Childhood and Adolescence, University Medical Center Hamburg-Eppendorf, Germany; German Center for Addiction Research in Childhood and Adolescence, University Medical Center Hamburg-Eppendorf, Germany; German Center for Addiction Research in Childhood and Adolescence, University Medical Center Hamburg-Eppendorf, Germany; German Center for Addiction Research in Childhood and Adolescence, University Medical Center Hamburg-Eppendorf, Germany

**Keywords:** Psychological stress, COVID-19 lockdown, adolescents, parents, risk factors

## Abstract

**Background:**

COVID-19 lockdown measures imposed extensive restrictions to public life. Previous studies suggest significant negative psychological consequences, but lack longitudinal data on population-based samples.

**Aims:**

We aimed to prospectively identify increased psychological stress and associated risk factors in parent–child dyads.

**Method:**

We conducted a prospective, observational online study on a representative German sample of 1221 adolescents aged 10–17 years and their parents. Psychological stress and psychosocial variables were assessed before the pandemic (baseline) and 1 month after the start of lockdown (follow-up), using standardised measures. We used multilevel modelling to estimate changes in psychological stress, and logistic regression to determine demographic and psychosocial risk factors for increased psychological stress.

**Results:**

The time of measurement explained 43% of the psychological stress variance. Of 731 dyads with complete data, 252 adolescents (34.5%, 95% CI 31.0–37.9) and 217 parents (29.7%, 95% CI 26.4–33.0) reported a significant increase in psychological stress. Baseline levels were lower than in dyads without increased psychological stress. Risk factors for increased psychological stress included sociodemographic (e.g. female parents, severe financial worries) and emotion regulation aspects (e.g. non-acceptance of emotional responses in parents, limited access to emotion regulation strategies in adolescents), explaining 31% of the adolescent (Nagelkerke *R*^2^ = 0.31) and 29% of the parental (Nagelkerke *R*^2^ = 0.29) model variance.

**Conclusions:**

This study is the first to prospectively show an increase in psychological stress during COVID-19 lockdown in a representative family sample. Identified demographic and psychosocial risk factors lead to relevant implications for prevention measures regarding this important public health issue.

The COVID-19 pandemic represents a major crisis that threatens physical and mental health. It places significant psychological burden on societies and individuals across the world.^1^ In the first weeks of the pandemic, many countries adopted public measures based on social distancing, to slow down the human-to-human spread of the novel virus. In mid-March 2020, the German Government legislated the nationwide closure of schools, child care facilities, sports venues and public playgrounds, along with the imposition of massive contact restrictions (termed ‘lockdown’).^2^ By that time, Europe was the epicentre of the pandemic. By mid-April, 977 569 cases and 84 607 COVID-19-associated deaths were reported in Europe. The most cases occurred in Spain (*n* = 172 541), Italy (*n* = 162 488) and Germany (*n* = 127 584). Among these countries, the death rate was lowest in Germany at that time (2.56%, compared with 12.97% in Italy and 10.46% in Spain).^3^ Case fatality rates could be shown to positively correlate with fear of the disease.^4^ Besides fear of death, severe lifestyle transformations, including decreased social contacts and upended daily structures, were associated with higher depression and anxiety rates in adolescents.^5^ This is supported by studies from previous virus outbreaks that indicate containment measures, such as quarantine, isolation and social distancing, can have extensive negative consequences for mental health.^6^ Moreover, the resulting high-density conditions (i.e. household crowding) are known to be particularly stressful and even traumatising to a significant proportion of children and parents.^2,6,7^

Initial studies support the notion of the COVID-19 pandemic being perceived as a stressful experience on a population level.^8^ One cross-sectional study identified 38% of the Italian general population as having experienced significant distress during early lockdown.^9^ Psychosocial conditions of children and their parents have long played a subordinate role in the public debate during the COVID-19 pandemic.^1,10^ To the best of our knowledge, no studies are available that have investigated psychological stress before and during the COVID-19 pandemic in representative family samples. Although stress increase in populations facing large-scale stressful events seems to be self-evident, such an approach is necessary to better understand the effects of an event unpredictable in duration.^1,6,10^ Assessing the subjective appraisal of threatening or challenging events in light of available coping resources (individually perceived stress), rather than life events *per se* (objective stress), could be shown to be superior in terms of predicting health and health-related outcomes.^11^ Global stress perception thus depends on contextual and personal factors, such as gender; age; educational, economical and occupational background;^12^ emotion regulation capabilities^13^ and parental self-efficacy.^14^

## Aims

To better identify those particularly vulnerable and thus in need of future targeted preventive action, this study aimed to assess prospective levels and potential risk factors of psychological stress in adolescents and their parents, from the general population in Germany. We relied on standardised, longitudinal subjective ratings of psychological stress before and during the COVID-19 pandemic, and expected that (a) a relevant proportion of adolescents and/or parents will report an increase in psychological stress during COVID-19 lockdown compared with pre-COVID-19 pandemic baseline measures; and (b) increases in psychological stress can be predicted by a prespecified set of sociodemographic variables, such as gender and financial worries, as well as potentially modifiable family-related and stress coping-associated factors, such as emotional dysregulation and behavioural avoidance (i.e. procrastination).

## Method

### Participants and procedure

A representative sample of 1221 German adolescents aged between 10 and 17 years, and 1221 respective parents, were included in this observational study, using an online battery of questionnaires (*N* = 2242). Of those, 824 parent–child dyads (67.5%) consented to join the follow-up measurement (*n* = 1648). Representativity was assured in terms of proportions of gender, age and region of residence by using a random sampling method from the well-established German market research and opinion polling company forsa Gesellschaft für Sozialforschung und statistische Analysen mbH (for details see the study by Paschke et al,^15^ and Supplementary Methods and Supplementary Fig. 1 in Supplementary Appendix 1, available at https://doi.org/10.1192/bjo.2021.49). The baseline data was collected as part of a large online survey on psychological stress and media usage in families on a population basis, and supplemented by follow-up measurements at approximately half-year intervals. The first interim results of an ongoing longitudinal study are presented here. The observational period was set between 13 and 27 September 2019 for baseline, and between 20 and 30 April 2020 for the first follow-up assessment, 1 month after the start of the German COVID-19 lockdown.

The authors assert that all procedures contributing to this work comply with the ethical standards of the relevant national and institutional committees on human experimentation and with the Helsinki Declaration of 1975, as revised in 2008. All procedures involving humans were approved by the Local Psychological Ethics Committee of the University Medical Center Hamburg-Eppendorf (ethical approval number LPEK-0145). Informed consent was obtained from all participants.

### Measures and outcome

We collected sociodemographic data on gender, age, place of residence, educational and first-generation migration background, parental partnership status and the number of underaged children in the household at baseline. Occupation (‘Which of the following descriptions of your occupation most closely applies to you?’ – example answers: ‘not employed’, ‘job-seeking’ versus ‘part-time employed’, ‘fully employed’), school attendance (‘What are you currently doing? Are you still in school, are you in training, are you employed or what else do you do?’) and financial worries (‘Which of the following statements about your financial situation applies most to you?’ – ‘I often worry about paying my bills’, ’ I/my child often have to do without something because I have to limit myself financially’ versus ‘I have enough money for everything I need’, ‘I have enough money to save something each month’) were measured by single items. Moreover, confidence in parenting was assessed with the validated nine-item Parental Self-efficacy Questionnaire (Fragebogen zur Selbstwirksamkeit in der Erziehung).^14^ Adolescents’ and parents’ emotion regulation problems were assessed by the short form of the Difficulties in Emotion Regulation Scale,^16^ a standardised 18-item tool organized into six subscales (non-acceptance of emotional responses, lack of emotional clarity, difficulties engaging in goal-directed behaviour under unpleasant emotions, impulse control difficulties, limited access to emotion regulation strategies and [lack of] emotional awareness). Behavioural avoidance was measured with the four-item Procrastination Questionnaire for Students (Prokrastinationsfragebogen für Studierende).^17^ This questionnaire was initially validated in German university students via online questionnaire. Because of the simple item structure with a five-point Likert-scale response ((almost) never to (almost) always) and its relation to study assignments (e.g. ‘I put off starting tasks until the last moment’), it has been frequently applied to high school students in clinical settings. This is supported by an excellent internal consistency for the present adolescent sample (Cronbach's *α* = 0.90). Moreover, during follow-up, parents and adolescents were asked whether they mainly stayed at home during COVID-19 lockdown.

The change in psychological stress from baseline to follow-up measurement was the primary outcome assessed by the Perceived Stress Scale (PSS-4).^11^ This four-item instrument has been validated in large international samples,^12^ including adolescents,^18,19^ and was applied at baseline and follow-up. Participants rated how frequently they have appraised their life as unpredictable, uncontrollable and overloading within the previous month. Likert-scale responses were summed up to a score of 0–16, with higher scores indicating higher general stress. A score of ≥ 8 could be referred to as elevated.^20^

### Statistical analysis

Absolute and relative frequencies were computed together with 95% confidence intervals for categorial variables, and mean values with s.d. for metric variables. Sociodemographic and psychological characteristics of the baseline and the follow-up adolescent and parental sample were compared with *χ*^2^- and *t*-tests for categorial/ metric variables, to account for the sample attrition. Equivalence testing was performed to compare mean baseline PSS-4 scores with the literature by calculating two one-sided tests (TOST; for a detailed description refer to the Supplementary Methods in Supplementary Appendix 1). To investigate the potential change of mean PSS-4 scores, paired-sample *t*-tests were computed. Effect sizes were calculated by Cohen's *d* for repeated measures (*d_rm_*).

Pearson correlation tests were used for PSS-4 values of parents and adolescents at baseline and follow-up. Correlation values were statistically evaluated using a *z*-test and Cohen's *q* for effect size estimation.

PSS-4 values of adolescents and corresponding parents were estimated in a multilevel model, with the measurement time point as an independent variable. Random effects were accounted for by considering participants and their nesting within parent–child dyads. To reveal the effect of between-group variance resulting from measurements before and during the COVID-19 pandemic, the intraclass correlation coefficient was calculated.

Based on the individual's change in psychological stress, the samples of adolescents and parents were each divided into two groups: group 1 showed an increase in psychological stress by >3 points (1 s.d.) and group 2 showed no increase in psychological stress. The sociodemographic characteristics and total scores of the psychological measures for these groups were compared by *χ*^2^- and *t*-tests (categorial/metric variables) and multivariate analysis of variance (psychometric variables), with separate *post hoc* Scheffé tests for adolescents and parents. Effect sizes were computed with Cramer's *V* (categorial variables) and Cohen's *d* (metric variables) for independent measures.

Logistic regression analyses were used to determine risk factors for increased psychological stress in adolescents and parents during COVID-19 lockdown, controlling for potential age and baseline psychological stress effects. Respectively, the sociodemographic and psychological baseline measures, time spent at home during COVID-19 lockdown and an increase in psychological stress (yes/no) of the corresponding parent/child were simultaneously entered into multivariate regression models, with age and PSS-4 baseline as covariates after dichotomising. Model reduction was performed by backwards elimination using the Akaike information criterion. Adjusted odds ratios were estimated based on the final model (for further details refer to the Supplementary Methods in Supplementary Appendix 1).

All statistical analyses were performed with the software package R version 4.0.2 for Windows.^21^

## Results

### Sample characteristics

A comparison between the baseline and follow-up sample, regarding sociodemographic factors and psychometric responses to account for potential attrition effects, did not reveal any significant differences (Supplementary Table 1 in Supplementary Appendix 1). Of the 824 dyads that participated in the baseline and the follow-up measurements, 731 sufficiently completed the PSS-4 and could be included in the family-based analyses (Supplementary Fig. 1 in Supplementary Appendix 1).

Table 1 shows the sample characteristics. The adolescent sample was 46.4% female. The adolescents’ mean age was 13.06 (s.d. 2.40). The adolescents showed mean PSS-4 values of 5.53 (s.d. 3.02) before the pandemic and 6.93 (s.d. 3.14) during COVID-19 lockdown. A paired *t*-test revealed a clinically significant increase in psychological stress in adolescents (*t*(823) = 11.44, *P* < 0.001, Cohen's *d_rm_* = 0.41). The parental sample was 50.9% female. The parents’ mean age was 46.5 (s.d. 8.0). The parents showed mean PSS-4 values of 5.33 (s.d. 2.98) before the pandemic and 6.33 (s.d. 2.99) during COVID-19 lockdown. Comparable with the adolescents, a paired *t*-test showed a clinically significant increase in psychological stress in the parental sample (*t*(823) = 9.13, *P* < 0.001, Cohen's *d_rm_* = 0.32).

**Table 1:** Sociodemographic sample description

Variables/categories	Follow-up sample, *n* (%) or mean (s.d.; range)
Adolescents	824
Gender	
Male	442 (53.6)
Female	382 (46.4)
Age, years	13.06 (2.4; 10–17)
(Prospective) school-leaving certificate^a^	
No/low educational degree^b^	30 (3.8)
Middle or higher educational degree^c^	762 (96.2)
High school student^d^	
Yes	763 (92.7)
No^e^	60 (7.3)
Parents	824
Gender	
Male	405 (49.2)
Female	419 (50.9)
Age, years	46.5 (8.0; 28–75)
Highest educational level^f^	
Low education^g^	73 (8.9)
Middle or high education^h^	750 (91.1)
Occupation	
Full-time or part-time employment	733 (89.0)
No employment	91 (11.0)
Relationship status^i^	
Single	63 (7.7)
Partnership	759 (92.3)
Parent–child dyad	
Number of underaged children in household^j^	1.7 (0.83; 1–13)
Place of residence	
Urban living	440 (53.4)
Rural living	384 (46.6)
Financial worries^k^	
Yes	33 (4.1)
No	782 (96.0)
First-generation migration background^l^	
Yes	36 (4.4)
No	787 (95.6)

a. No answer, *n* = 32.

b. No, special school (*Förderschulabschluss*) or lower school certificate (*Hauptschulabschluss*).

c. Secondary school certificate (*Realschulabschluss*) to university entry qualification (*Abitur*).

d. No answer, *n* = 1.

e. In voluntary service, apprenticeship, national service or job-seeking.

f. No answer, *n* = 1.

g. No or lower school certificate (*Hauptschulabschluss*).

h. Secondary school certificate (*Realschulabschluss*) to doctor's degree (PhD).

i. No answer, *n* = 2.

j. No answer, *n* = 4.

k. No answer, *n* = 9.

l. No answer, *n* = 1.

The parental PSS-4 baseline value was equivalent to the average score of large samples of 37 451 European participants (mean difference of 0.1, TOST 90% CI −0.08 to 0.28).^14^ The adolescent baseline score was 1.2 points lower than the PSS-4 total score of a large sample of 29 388 British adolescents (mean difference of 1.2, TOST 90% CI 1.01–1.39).^15^ A younger British sample showed an equivalent value (mean difference of 0.1, TOST 90% CI −0.17 to 0.23).^16^ Please refer to the Supplementary Results in Supplementary Appendix 1 for further details.

### Psychological stress before and during the pandemic

**Fig. 1:**
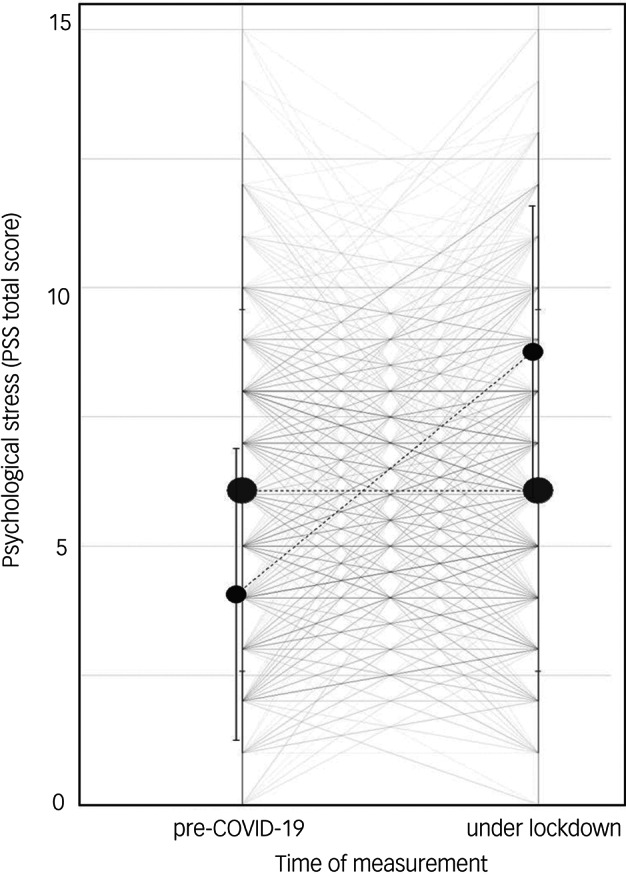
Multilevel analysis: trajectories of psychological stress before the COVID-19 pandemic and under lockdown measures. The individual trajectories of all adolescents and parents are shown, together with means and 95% confidence intervals of individuals belonging to the increased psychological stress group and non-increased psychological stress group, in reference to the two measurement points. The size of the dots reflects the group size. Accordingly, smaller dots represent the increased psychological stress group and larger dots represent the non-increased psychological stress group.

PSS-4 values of adolescents and their respective parents showed moderate correlations at baseline (*r* = 0.38, *P* < 0.001) and slightly higher (moderate) correlations at follow-up (*r* = 0.45, *P* < 0.001; comparison: *z* = 3.24, Cohen's *q* = 0.17). A multilevel analysis of the PSS-4 values of all participants, taking into account the parent–child dyads, revealed an intraclass correlation coefficient value of 0.43 (Supplementary Table 2 in Supplementary Appendix 1). This indicates that 43% of the PSS-4 variance is explained by the time of the measurement (follow-up 1 month after start of COVID-19 lockdown versus baseline 7 months earlier). The different trajectories of psychological stress development are visualised in Fig. 1.

#### Adolescents

A total of 252 adolescents (34.47%, 95% CI 31.03–37.92) reported an increase in psychological stress from baseline to follow-up. These adolescents showed significantly lower PSS-4 values before the pandemic (4.36 *v*. 6.81, *t*(609.54) = −12.79, *P* < 0.001, *d* = 0.93) and significantly higher values during COVID-19 lockdown, compared with adolescents who did not experience increased stress (9.15 *v*. 6.22, *t*(537.48) = 14.46, *P* < 0.001, *d* = 1.10), with large effect sizes.

#### Parents

A total of 217 parents (29.69%, 95% CI 26.37–33.00) experienced an increase in psychological stress during COVID-19 lockdown. They showed significantly lower PSS-4 values before the pandemic (3.74 *v*. 6.00, *t*(546.91) = −11.13, *P* < 0.001, *d* = 0.80) and significantly higher values during COVID-19 lockdown (8.33 *v*. 5.51, *t*(427.98) = 13.41, *P* < 0.001, *d* = 1.06), compared with parents who did not experience increased stress, again with large effect sizes.

A detailed comparison on sociodemographic and psychological parameters, including results of the multivariate analyses of variance and *post hoc* tests (psychometric variables) between groups with and without increased psychological stress, can be found in Supplementary Appendix 1 (Supplementary Results and Supplementary Tables 3 and 4).

### Risk factors for increase in psychological stress

#### Adolescents

Six out of fifteen predictors and the covariate baseline psychological stress (covariate age excluded) stayed in the logistic regression model after backwards elimination for the adolescents (Supplementary Table 5 in Supplementary Appendix 1). The remaining variables explained 31% of the final adolescent model variance (Nagelkerke *R*^2^ = 0.31). Significant adolescent risk factors for increased psychological stress included financial worries (adjusted odds ratio 2.13, 95% CI 1.29–3.51), increased psychological stress of the corresponding parent (adjusted odds ratio 2.33, 95% CI 1.56–3.49), procrastination (adjusted odds ratio 2.10, 95% CI 1.27–3.48), limited access to emotion regulation strategies (adjusted odds ratio 2.01, 95% CI 1.21–3.35) and mainly staying at home during COVID-19 lockdown (adjusted odds ratio 1.65, 95% CI 1.08–2.50). High emotional awareness served as a protective factor for adolescents, with an adjusted odds ratio of 0.47 (95% CI 0.29–0.77).

#### Parents

Ten out of nineteen predictors and the two covariates (baseline psychological stress and age) stayed in the logistic regression model after backwards elimination for the parents (Supplementary Table 6 in Supplementary Appendix 1). The remaining variables explained 29% of the final parental model variance (Nagelkerke *R*^2^ = 0.29). Significant parental risk factors for increased psychological stress could be identified, including female gender (adjusted odds ratio 1.86, 95% CI 1.29–2.76), partnership status (adjusted odds ratio 2.78, 95% CI 1.10–6.98), urban living (adjusted odds ratio 1.45, 95% CI 1.00–2.10), financial worries (adjusted odds ratio 1.86, 95% CI 1.08–3.19), increased psychological stress of the corresponding adolescent (adjusted odds ratio 1.59, 95% CI 1.09–2.33), procrastination (adjusted odds ratio 1.70, 95% CI 1.00–2.89) and non-acceptance of emotional responses (adjusted odds ratio 3.02, 95% CI 1.78–5.13).

**Fig. 2:**
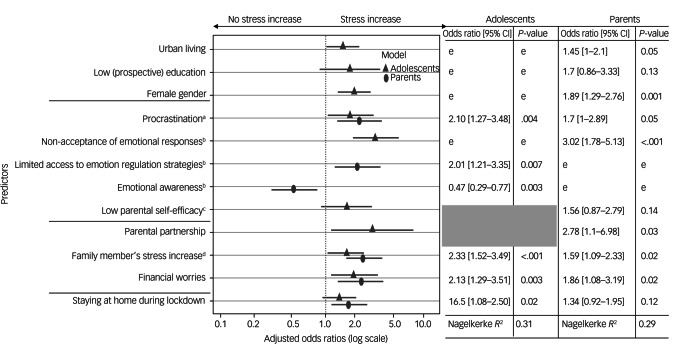
Adjusted odds ratios with 95% confidence intervals for predictors of increased psychological stress during COVID-19 lockdown measures. The shaded area represents variables that were not assessed in the adolescent sample, and therefore could not be considered as predictors for the adolescent model. Adjusted odds ratio was according to the logistic regression model, with covariates of baseline stress level and age. Reduced logistic regression adolescent model: covariate baseline stress level odds ratio 0.62, 95% CI 0.57–0.68, *P* < 0.001. Reduced logistic regression parental model: covariate baseline stress level odds ratio 0.65, 95% CI 0.60–0.71, *P* < 0.001; covariate parental age odds ratio 1.02, 95% CI 0.99–1.04, *P* = 0.16. ^a^Assessed with the Procrastination Questionnaire for Students (Prokrastionationsfragebogen für Studierende). ^b^Assessed with the short form of the Difficulties in Emotion Regulation Scale. ^c^Assessed with the Parenting Self-efficacy Questionnaire (Fragebogen zur Selbstwirksamkeit in der Erziehung). ^d^Assessed with the Perceived Stress Scale (PSS-4). ^e^Not included in final model after backwards elimination of predictors.

All adjusted odds ratios are visualised in Fig. 2.

## Discussion

To our knowledge, this is the first study to investigate psychological stress within a representative sample of adolescents and their parents during COVID-19 lockdown, compared with pre-COVID-19 conditions, in a longitudinal design. A total of 43% of the psychological stress variance in the parent–child dyads could be explained by the time of measurement (September 2019 versus April 2020). We found that about a third of the adolescents and parents showed a significant increase in psychological stress during COVID-19 lockdown. Although increased psychological stress seems to be a normal human reaction to the COVID-19 pandemic,^6^ this finding appears meaningful from a public health perspective because psychological stress is a major predisposing factor for health problems, including (neuro-)psychiatric sequelae.^22,23^ This is of particular importance in adolescents, given their increased vulnerability to stress and psychopathology in a period of substantial neurobiological and psychological changes.^24^ The finding builds on initial cross-sectional studies from Germany,^25^ Italy^9^ and China,^26,27^ showing that about a quarter of German, a third of Italian and up to half of Chinese adults reported feelings of mental distress during the initial stage of the COVID-19 pandemic.

Interestingly, baseline psychological stress level could be identified as a major factor that needs to be considered when investigating potential stress increase. Adolescents and parents who experienced a significant increase in psychological stress had lower baseline stress levels than those who did not experience increased psychological stress during COVID-19 lockdown. This might suggest that the latter group has better developed stress-coping mechanisms than those with lower baseline stress.^28^ On the other hand, albeit hypothetical at the present stage of research, COVID-19 lockdown consequences, such as school closures and home office implementation, might actually be stress-reductive among a subsample of adolescents and parents. Correspondingly, family cohesion and better family adjustment could be promoted, and school-related problems (like low academic achievement, experiencing social pressure or being bullied) might decrease.^29^

Furthermore, this is the first study to prospectively identify sociodemographic, family-associated and psychological risk factors for increased psychological stress during COVID-19 lockdown in adolescents and their parents, controlling for potential age and baseline stress effects. The sociodemographic risk factors include parental female gender, urban living, parental partnership status, family's financial worries and the time adolescents spent at home during COVID-19 lockdown. A higher increase in psychological stress in mothers was about twice as likely over time. This is consistent with prior research,^6,9,25,30^ and could be related to gender-specific responses toward environmental stressors, the burden associated with additional role demands (e.g. regarding home schooling, psychological family support, economic and job-related aspects) and a higher vulnerability of women toward stress-related disorders.^31^ In their recent review, Connor et al analysed health-related international public and governmental databases on severe acute respiratory syndrome (SARS) in 2003, Ebola virus disease in 2014, Zika virus disease in 2016 and COVID-19.^31^ They found that women experience higher ‘caregiver burden’ and less access to the healthcare system during lockdowns, because of reduced social support, isolation and increased negative economic effects of the pandemic compared with men. Urban living – a mental health challenge^32^ – could be identified as a moderate risk factor for parental increase in psychological stress (adjusted odds ratio of 1.45). One interesting finding, standing in partial contradiction to prior literature, is the effect of partnership: although some publications do not suggest an effect of marriage status,^6,9^ one study reported higher distress in multi-person compared with single-person households.^30^ In line with the latter, we could relate being in a partnership to almost three times higher odds for increased psychological stress in parents. Evidence from previous studies supports our finding, suggesting that containment-like measures under pandemic circumstances may create a highly stressful condition for families and children.^33^ Although the majority of the German population was not under a mandated quarantine, 72% of our adolescent sample reported having spent most of their time at home during COVID-19 lockdown. These adolescents were 65% more likely to report an increase in psychological stress compared with adolescents that stated otherwise. Increased time at home, particularly under high-density conditions, is associated with a lack of physical activity and increased media use.^34^ It is consequently a risk factor for reduced well-being, symptoms of psychological disorders such as anxiety and depression, and conduct problems, especially in low-income populations.^35^ Moreover, fear of socioeconomic deprivation during lockdown can cause psychological stress.^6^ Low-income families may be threatened more existentially by COVID-19 lockdown-imposed consequences to their working lives. These considerations are in line with our finding that parents and adolescents with financial worries were 1.86 (parents) to 2.13 (adolescents) times more likely to experience an increase in psychological stress. Moreover, our results indicate significant family-related risk factors: significant associations between increased psychological stress of adolescents and the corresponding parent were found (adjusted odds ratio of 1.59 for parents, to 2.33 for adolescents). This is consistent with the repeatedly confirmed mediating influence of parent-based variables on psychological symptoms in children and adolescents.^24^

From a psychological perspective, problems in emotion regulation could be identified as risk factors of increased psychological stress. Adolescents with limited access to emotion regulation strategies were twice as likely to experience increased psychological stress, and parents with a low acceptance of emotional responses were three times as likely to experience increased psychological stress. Moreover, procrastination – an avoidance-based, short-term emotion regulation strategy^36^ – could be identified as a risk factor for adolescent (adjusted odds ratio of 2.10) and parental increased psychological stress (adjusted odds ratio of 1.70). In contrast, emotional awareness (the appraisal and recognition of one's own feelings) could be identified as a potentially modifiable psychological protective factor against increased psychological stress in adolescents (adjusted odds ratio of 0.47). Aside from external factors, cognitive appraisal processes determine whether and how much a situation is perceived as stressful, triggering coping strategies such as emotion regulation to reduce negative feelings.^37^ Considering intraindividual difficulties in emotion regulation can help to understand interindividual differences in psychological stress during COVID-19 lockdown measures, thereby providing potential anchors for prevention approaches at individual and family levels. These might include elements of established programs such as mindfulness-based cognitive therapy, dialectical behavioural therapy or acceptance-based behavioural therapy.^38^ Moreover, activity-based interventions, such as yoga, sports, play and creative arts, have been shown to improve mental health, and might be promising in providing helpful offers for adolescents to spend time outside the home without violating restriction rules.^39^ Online interventions might be a useful new method to support mental health,^20^ especially during a time where personal contacts are limited.

### Strengths and limitations

The study has numerous strengths, including the use of prospective data with an assessment just before the pandemic and during COVID-19 lockdown; a large study population representative regarding gender (most of the studies available have included a significant higher proportion of female respondents), age and region of living; the data of parent–child dyads; information on potential risk factors before the COVID-19 pandemic and the variety of standardised subjective ratings to collect data on psychological stress and potential psychological risk factors. However, several limitations of this study need to be considered.

First, although sample representativity was warranted in terms of age, gender and place of residence, representativity in other respects might have been reduced because of the data collection procedure. In this respect, the sample only includes households with sufficient knowledge of the German language, so families with an immigrant background might not have been properly taken into account. In addition, around 5% of German households do not have internet access^40^ and could not be included in this study. Moreover, although online questionnaires are highly valued for cost-effectiveness in large epidemiological studies, missing data is a common problem, especially when investigating dyads and children. Accordingly, 113 parent–child dyads had to be omitted from subsequent analysis, which might have decreased representativity. In addition, all participants were asked to complete the questionnaire independently, but the influence of others cannot be excluded.

Second, although our study revealed a significant increase in psychological stress in families on a population level, and identified risk factors during the first German COVID-19 lockdown, it could not explain the individual circumstances that may have preceded these increases. In fact, we cannot rule out the possibility that other factors or unknown third variables during the survey interval contributed to the variance in the prospective psychological stress change. Moreover, we cannot conclude on the individual factors that actually led to the observed increases in psychological stress, because of the restricted number of items in an online survey, such as the number of critical life events, family members affected by COVID-19 and lockdown-induced economic changes. This was not the primary aim of the study and is a complex issue, as research shows that youth stress responsiveness is a highly volatile construct, affected by numerous family-related, developmental and other biopsychosocial aspects.^41^ Nevertheless, based on prior research, we can assume that the COVID-19 pandemic was a significant source of stressors in the current sample, allowing for conclusions on a population level.^42^ Finally, it should be kept in mind that the reported results are from an ongoing study. Thus, the added value of prospective studies by means of multiple measurement points has not yet been fully exploited.

### Clinical relevance and future research

This prospective observational study is the first to report on the increase in psychological stress in adolescents and their parents, as well as associated demographic and psychosocial risk factors in a representative German family sample during COVID-19 lockdown, compared with pre-COVID-19 conditions. It thus adds knowledge to the current evidence base on the psychological impact of this worldwide pandemic. Our findings have several implications for clinicians, researchers, policy makers and social actors. As the COVID-19 pandemic continues, psychological stress and its corresponding threat to mental health should be of greater consideration among public health authorities. Besides protecting the population from the disease, adolescents and their parents – especially mothers – could benefit from targeted psychological stress prevention measures. The identification of sociodemographic, family-associated and potentially modifiable psychological risk factors, such as capacity for adaptive coping, provides decision makers with meaningful insights for public health action.

Future studies should focus on the detailed investigation of factors explaining individual increased psychological stress and resiliency. Although the present study could not identify adolescents’ age as a significant covariate regarding psychological stress, future research should investigate age aspects in more detail by including younger children and their parents. Moreover, cross-cultural studies should enhance knowledge on the comparability of the presented findings. Psychological stress variability during the course of the pandemic should be investigated by follow-up measurements.

## Data Availability

The data that support the findings of this study are available from the corresponding author, K.P., upon reasonable request, after all results of the parent–child survey have been published.

## Supplementary material

For supplementary material accompanying this paper visit http://dx.doi.org/10.1192/bjo.2021.49. click here to view supplementary material
