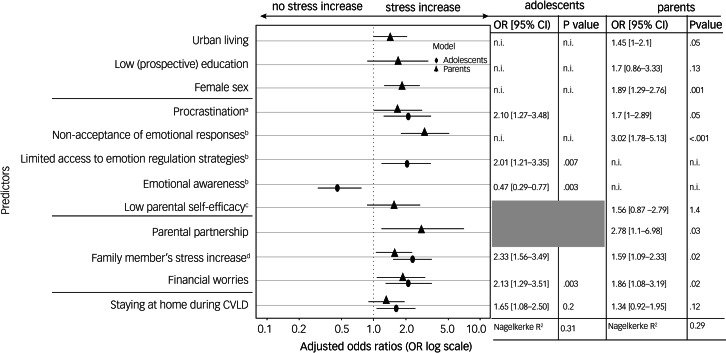# Risk factors for prospective increase in psychological stress during COVID-19 lockdown in a representative sample of adolescents and their parents – ERRATUM

**DOI:** 10.1192/bjo.2021.1038

**Published:** 2021-12-17

**Authors:** Kerstin Paschke, Nicolas Arnaud, Maria Isabella Austermann, Rainer Thomasius

**Keywords:** Psychological stress, COVID-19 lockdown, testing, adolescents, parents, risk factors, corrigendum

This article was published with an error in Figure 2 where the symbols for adolescents and parents were accidentally switched around. The publisher apologises for this mistake and the correct version of the figure is below.

**Fig. 2:**