# Supplementary material for: Risk factors for prospective increase in psychological stress during COVID-19 lockdown in a representative sample of adolescents and their parents
Source: BJPsych Open. 2021 May 3;7(3):e94. doi: 10.1192/bjo.2021.49 (PMC8111205; doi:10.1192/bjo.2021.49)
Supplement: Supplementary file 1 [file S2056472421000491sup001.docx]

**Risk factors for prospective psychological stress increase during coronavirus lockdown in a representative sample of adolescents and their parents**

**Supplementary Material**

**Supplementary Methods.**

**Supplementary Results.**

**Supplementary References.**

**Supplementary Tables 1-6.**

**Supplementary Figure 1.**

**Supplementary Methods**

**Participants and recruitment**

Initially, 23 736 representative households of a cluster with adults aged 28 to 75 years were contacted via e-mail. These households belong to the forsa.omninet panel including adults and adolescents of 14 years and above (1). Of 12 427 respondents (52.36%), 1733 (13.9%) fulfilled the inclusion criteria by reporting having children in the age of interest. In households with more than one child in the required age, the minor with the most recent birthday was invited for participation. 1221 parents and 1221 adolescents provided full necessary information at the baseline data collection (70.5% of respondents fulfilling the inclusion criteria). Of those, 824 parent-child dyads (67.5%, N=1648) consented to join the follow-up measurement (Supplementary Figure 1). Each participant gave his/her informed consent prior to enrolment. Adolescents and their parents were asked to complete the online questionnaires subsequently and independently.

Representativity could be ensured regarding age, sex, and region of residence by the underlying random-sampling method and according to the federal population statistics available at the time of recruitment (microcensus data 2017) (2). Proportions regarding education, occupation, number of underaged children per household, and financial worries were comparable to other representative samples (3,4). The proportion of parents without partnership (“singles”) was lower than the proportion of single parents (ca. 8%) according to microcensus data (18%) (5). Although, both aspects cannot be compared directly, since in the current survey, parents were asked if they are in a partnership regardless of whether they live in a common household and share parenting responsibilities.

**Timeline and lockdown measures (January to April 2020)**

- January 2020: the WHO declared the COVID-19 outbreak to be a public health emergency of international concern;
- January 27: First COVID-19 case was confirmed in Germany;
- Issued on March 22 by the German government : Adoption of strict and large scale social distancing measures (“contact ban”) to (6):
  1. protect the health of the population and maintain the efficiency of the health system;
  2. mitigate the consequences for citizens, employees and companies;
  3. tackle the pandemic together with European and international partners(2)

Social distancing measures included:

- Maintaining 1.5-meter distance to others;
- Ban of public gatherings of more than two people, except for families and those who live together;
- Closure of restaurants, bars and cafés and businesses;
- Closure of schools and daycare facilities;

Notably, while in 14 of the 16 German states people were allowed to leave their homes at all times, this was not the case in Bavaria and Saarland.

- April 20: Shops up to 800 square meters are allowed to reopen;
- April 27: Obligation to wear face masks in public buildings, shops and public transport all across Germany

**Details of statistical analysis**

Data management and analytic strategies

Subjects with more than one missing value in their PSS-4 answer pattern were excluded from further analysis. This led to a total number of 93 excluded cases. Using the R package MICE (7), multiple imputations were carried out regarding missing values of the remaining 731 parental-adolescent dyads. This resulted in overall replacements of 0.17 to 1.27% (adolescents baseline: 1.27% and follow-up: 1.16%; parents baseline: 0.17% and follow-up: 0.31%). According to missing data on single items or standardized-scale items of more than one third (DERS subscales, PFS, FSW), 68 parents and 91 adolescents had to be excluded before model building. Remaining missing standardized scale items were multiply imputated leading to replacements of 0.06% to 2.39%.

The baseline PSS-4 score of the analyzed parents and adolescents were compared with PSS-4 scores from the literature using mean differences. Equivalence testing was realized by calculating two one-sided tests (TOST), following literatures’ suggestions for meaningful upper and lower bounds of Δ=±0.3 for 90% desired power and an α of 0.05 (8,9). TOST for independent means with unequal sample sizes and unequal variances were computed with the R package TOSTER (8). Equivalence would be assumed if the mean difference was within the equivalence bounds, the equivalence test reached significance, and the null-hypothesis test was non-significant.

Sociodemographic, family, and psychological baseline measures as well as the time spent at home during coronavirus lockdown served as predictors and covariates leading to a total number of 15 predictors and 2 covariates for the adolescent model and 19 predictors and 2 covariates for the parental model. Included variables were dichotomized to better correspond to the mathematical rationale of odds ratios: (prospective) educational level (low vs. middle/high), occupation (employed vs. non-employed)/school attendance (yes vs. no), urban living (yes vs. no), psychological stress increase of the corresponding family member having participated in the survey (according to stress group 2), family migration background, parental partnership, number of underaged children in household, and severe financial worries (all yes vs. no), parental self-efficacy (individual FSW sum score 1 SD below average, parental model only), emotion regulation (average sum scores 1 SD above average regarding DERS subscales non-acceptance, non-clarity, strategies, impulsiveness, goals, and 1 SD below-average in inversed-coded scale awareness), procrastination (PFS-sum scores 1 SD above average), as well as staying at home during coronavirus lockdown (yes vs. (rather) no). Age and baseline stress level (PSS-4) served as covariates. Adjusted odds ratios were computed for each variable included in the backwards logistic regression model.

Effect sizes

Effect sizes for correlation comparisons were estimated by Cohen’s q with q ≥ 0.10 symbolizing small, q ≥ 0.30 medium-sized, and q ≥ 0.50 large effects. Effect sizes to compare the PS groups were computed using Cramer’s V (categorial variables) and Cohen’s d (metric variables) for independent measures with the following interpretation of the absolute values: Cramer’s V > 0.25 very strong, > 0.15 strong, > 0.1 moderate, and > 0.05 weak effect; Cohen’s d > 0,8 large, > 0,5 medium, > 0,2 small effect (10).

Internal Consistency

The internal consistency of the psychometric scales was calculated using Cronbach’s α and McDonald’s ω. Coefficients of Cronbach’s α ≥ 0.60 (11) and McDonald’s ω ≥ 0.70 were regarded as acceptable (12).

**Supplementary Results**

**Internal Consistency**

Perceived Stress Scale (PSS-4)

The internal consistencies of the applied PSS-4 were acceptable for the adolescents (baseline: Cronbach’s α=0.61, McDonald’s ω=0.76; follow-up: Cronbach’s α=0.63, McDonald’s ω=0.74) and the parents (baseline: Cronbach’s α=0.70, McDonald’s ω=0.80; follow-up: Cronbach’s α=0.70, McDonald’s ω=0.78). The Cronbach’s α values are comparable to those calculated on the basis of 37 451 adults (Cronbach’s α=0.74) (13) and 29 388 adolescents (Cronbach’s α=0.62) (14).

Parental Self-efficacy Questionnaire (FSW)

Cronbach’s α of the parental self-efficacy questionnaire FSW revealed a value of 0.79 and, thus, an acceptable internal consistency.

Procrastination Questionnaire for Students (PFS-4)

Good to excellent internal consistency was suggested by a Cronbach’s α for the procrastination scale PFS (adolescents: 0.90, parents: 0.88).

Difficulties in Emotion Regulation Scale (DERS-SF)

Cronbach’s α of the DERS-SF total scale was 0.88 for the parents and 0.90 for the adolescents suggesting excellent internal consistency in our sample. Subscale values ranged between 0.64 and 0.86 for the parents (strategies: 0.78, non-acceptance: 0.70, impulsivity: 0.86, goals: 0.86, clarity 0.72, awareness: 0.64) and 0.67 to 0.90 for the adolescents (strategies: 0.79, non-acceptance: 0.71, impulsivity: 0.90, goals: 0.84, clarity 0.81, awareness: 0.67).

**Perceived baseline stress**

The parental average PSS-4 baseline score of 5.33 (s.d. = 2.98) was similar to the previously reported mean of 5.43 (s.d. = 2.96) based on a large sample of N = 37,451 European participants (13) (equivalence test: t(758.38) = -7.11, P < 0.001, mean difference = 0.1, TOST 90% CI -0.08-0.28; null-hypothesis test: t(758.38) = 0.90, P = 0.369). Based on the equivalence test and the null-hypothesis test combined, it can be concluded that the observed effect is statistically not different from zero and statistically equivalent to zero. No comparable sample exists for the adolescents. Our 10- to 17-year-old sample was slightly younger than a large British sample of 29,388 adolescents aged 11 to 16 years (14) (t(30210) = 6.13, P < 0.001). Their mean PSS-4 baseline score of 5.53 (s.d.=3.02) was about one point lower and not equivalent to the British mean score of 6.73 (s.d. = 3.17): equivalence test: t(770.55) = 2.40, P = 0.992, mean difference = 1.2, TOST 90% CI 1.01-1.39; null-hypothesis test: t(770.55) = 10.60, P < 0.001). However, the mean PSS-4 values of a (younger) British sample of 4320 11- to 12-year olds of 5.56 (s.d. = 2.95) were similar based on the equivalence and the null-hypothesis test results (15): equivalence test: t(980.44) = -7.19, P < 0.001, mean difference = 0.1, TOST 90% CI -0.17-0.23; null hypothesis test: t(980.44) = 0.249, P = 0.803.

**Comparison of increased and non-increased psychological stress groups**

**Adolescents.** A MANOVA of the psychometric variables with the adolescents belonging to the increased or non-increased PS group as dependent variable revealed significant differences with Pillai score(1,690) = 0.07, and F_approx_ (9,682) = 5.68, P < 0.001. Adolescent DERS sum scale values were significantly lower in the increased compared to the non-increased PS group at baseline with a small effect size suggesting slightly less problems in emotion regulation (37.50 vs. 40.30, t(535.8) = -3.36, P < 0.001; d = 0.26). All DERS subscales revealed slightly lower values in the increased than in the non-increased PS group. Parents of the adolescent increased PS group experienced significant higher PS levels under CVLD than those of the adolescent non-increased PS group (6.06 vs. 6.89, t(486.67) = -3.60, P < 0.001; d = 0.29) whereas no difference was found before the pandemic. Moreover, no differences were found in demographic and social variables (for details see Supplementary Table 3).

**Parents.** A MANOVA of the psychometric variables with the parents belonging to the increased or non-increased PS group as dependent variable revealed significant differences with Pillai score(1,719) = 0.07 and F_approx_ (10,710) = 5.06, P < 0.001. Parental DERS sum scale values were also significantly lower in the increased compared to the non-increased PS group at baseline indicating slightly lower problems in emotion regulation (31.58 vs. 34.07, t(477.62) = -3.30, P = 0.001; d = 0.25)). Four of the six DERS subscales (limited access to emotion-regulation strategies, lack of emotional clarity and awareness) revealed slightly lower values for the increased than for the non-increased PS group of parents. Whereas the children of parents of the PS increase group showed significant higher PS levels during CVLD (7.89 vs. 6.95, t(367.22) = 3.76, P < .001; d = 0.32), they did not differ from the children of parents of the PS non-increase group before the pandemic. No significant group differences were found regarding procrastination and parental self-efficacy. The proportion of mothers was significantly higher in the increased PS group than in the non-increased PS group (59.91% vs. 47.08%, χ^2^(1) = 9.54, P = 0.002; V = 0.12), so was the proportion of parents without partnership (8.58% vs. 3.24%, χ^2^(1)=5.86, P=.02; V=0.10). No group differences were observed within the other sociodemographic variables (Supplementary Table 4).

**Supplementary** **References**

1. forsa. https://www.forsa.de/1/methods/ (Accessed April 1, 2020).

2. https://www.destatis.de/EN/Home/_node.html (Accessed May 15, 2020).

3. https://www.destatis.de/DE/Themen/Gesellschaft-Umwelt/Bevoelkerung/Haushalte-
 Familien/Publikationen/Downloads-Haushalte/haushalte-familien-
 2010300177004.pdf%3F__blob%3DpublicationFile (Accessed May 15, 2020).

4. https://www.statistischebibliothek.de/mir/servlets/MCRFileNodeServlet/DEHeft_derivate_00053166/
 2010130177004_korr10022020.pdf (Accessed May 15, 2020).

5. https://www.destatis.de/DE/Presse/Pressekonferenzen/2018/Alleinerziehende/pressebroschuere-
 alleinerziehende.pdf?__blob=publicationFile (Accessed May 15, 2020).

6. Federal Ministry of Health. https://www.bundesgesundheitsministerium.de/coronavirus/chronik-coronavirus.html. Coronavirus SARS-CoV-2: Chronicle of measures taken so far [Coronavirus SARS-CoV-2: Chronik der bisherigen Maßnahmen] (Accsessed July 27, 2020).

7. Buuren S van, Groothuis-Oudshoorn K. mice: Multivariate Imputation by Chained Equations in R. *J Stat Softw.* 2011; **45**(3):1–67.

8. Lakens D. Equivalence tests: A practical primer for t-tests, correlations, and meta-analyses. *Soc Psychol
 Personal Sci*. 2017; **1**:1–8. https://doi.org/10.1177/1948550617697177.

9. Shishkina T, Farmus L, Cribbie RA. Testing for a lack of relationship among categorical
 variables. *Quant Meth Psych*. 2018; **14**(3), 167–79.
 https://doi.org/10.20982/tqmp.14.3.p167.

10. Cohen J. A power primer. *Psychol bull.* 1992; **112**(1):155–9, https://doi.org/10.1037//0033-2909.112.1.155.

11. Ursachi G, Horodnic IA, Zait A. How reliable are measurement scales? External factors with indirect influence on reliability estimators. *Procedia Econ Financ.* 2015;20:679–86.

12. Nunnally J. Psychometric theory. Mc Graw-Hill Publ Co. New York. 1978.

13. Vallejo MA, Vallejo-Slocker L, Fernández-Abascal EG, Mañanes G. Determining Factors for Stress Perception Assessed with the Perceived Stress Scale (PSS-4) in Spanish and Other European Samples. *Front Psychol.* 2018; **9**. https://doi.org/10.3389/fpsyg.2018.00037.

14. Demkowicz O, Panayiotou M, Ashworth E, Humphrey N, Deighton J. The factor structure of the 4-item Perceived Stress Scale in English adolescents. *Eur J Psychol Assess.* 2019*;* **36**:913-917. https://doi.org/10.1027/1015-5759/a000562.

15. Cartwright M, Wardle J, Steggles N, Simon AE, Croker H, Jarvis MJ. Stress and dietary practices in
 adolescents. *Health Psychol* 2003; **22**:362–9.

| **Supplementary Table 1.** Variable comparisons between baseline and follow-up sample. | | | |
| --- | --- | --- | --- |
| **Variables/Categories** | **Baseline sample**  **N (%)/mean (s.d.; range)** | **Follow-up sample**  **N (%)/mean (s.d.; range)** | **Statistical comparison**  **χ^2^/t (P value^a^)** |
| **Adolescents** | 1221 | 824 |  |
| **Gender** 0.004 (.95) | | | |
| Male | 658 (53.89) | 442 (53.64) |  |
| Female | 563 (46.11) | 382 (46.36) |  |
| **Age in years** | 13.04 (2.39; 10-17) | 13.06 (2.4; 10-17) | -0.19 (.85) |
| **(Prospective) school-leaving certificate**^b^ | | | 0.001 (.98) |
| No/low educational degree^d^ | 99 (8.48) | 66 (8.33) |  |
| Middle or higher educational degree^e^ | 1069 (91.52) | 726 (91.67) |  |
| **High school student^f^** |  |  | 0.00 (1.00) |
| Yes | 1132 (92.79) | 763 (92.71) |  |
| No^g^ | 88 (7.21) | 60 (7.29) |  |
| **Emotion regulation**^h^ |  |  |  |
| **Sum score** | 37.35 (14.42) | 37.13 (13.87) | 0.36 (.72) |
| Limited access to emotion-regulation strategies | 5.79 (3.05) | 5.80 (2.97) | -0.07 (.94) |
| Non-acceptance of emotional responses | 5.78 (3.00) | 5.80 (2.96) | -0.19 (.85) |
| Impulse control difficulties | 5.45 (3.20) | 5.41 (3.14) | 0.24 (.81) |
| Difficulties engaging in goal-directed  behavior under unpleasant  emotions | 6.72 (3.37) | 6.69 (3.23) | 0.21 (.83) |
| Lack of emotional clarity | 6.03 (3.08) | 5.99 (3.01) | 0.27 (.78) |
| Emotional awareness | 7.59 (3.39) | 7.43 (3.25) | 1.06 (.29) |
| **Procrastination^i^** | 10.87 (4.20) | 10.88 (4.16) | -0.01 (.99) |
| **Psychological baseline stress**^j^ | 5.59 (3.05) | 5.53 (3.02) | 0.48 (.63) |
| **Parents** | 1221 | 824 |  |
| **Gender** |  |  | 0.05 (.83) |
| Male | 593 (48.57) | 405 (49.15) |  |
| Female | 628 (51.43) | 419 (50.85) |  |
| **Age in years** | 46.21 (8.15; 28-75) | 46.46 (7.98; 28-75) | -0.69 (.49) |
| **Highest educational level**^k^ |  |  | 0.00 (1.00) |
| Low education^l^ | 106 (8.88) | 73 (8.87) |  |
| Middle or high education^m^ | 1088 (91.12) | 750 (91.13) |  |
| **Occupation** |  |  | 0.004 (.95) |
| Full-time or part-time employment | 1083 (88.77) | 733 (88.96) |  |
| No employment | 137 (11.23) | 91 (11.04) |  |
| **Relationship status**^n^ |  |  | 0.64 (.42) |
| Single | 107 (8.76) | 63 (7.66) |  |
| Partnership | 1114 (91.24) | 759 (92.34) |  |
| **Emotion regulation**^o^ |  |  |  |
| **Sum score** | 33.05 (10.33) | 32.93 (10.24) | 0.26 (.79) |
| Limited access to emotion-regulation strategies | 5.29 (2.51) | 5.28 (2.53) | 0.05 (.96) |
| Non-acceptance of emotional responses | 5.41 (2.50) | 5.33 (2.47) | 0.69 (.49) |
| Impulse control difficulties | 4.38 (2.14) | 4.32 (2.15) | 0.54 (.59) |
| Difficulties engaging in goal-directed  behavior under unpleasant  emotions | 6.33 (2.78) | 6.31 (2.78) | 0.10 (.92) |
| Lack of emotional clarity | 4.69 (2.08) | 4.71 (2.09) | -0.26 (.80) |
| Emotional awareness | 6.96 (2.67) | 6.96 (2.64) | -0.01 (.99) |
| **Procrastination**^h^ | 9.62 (3.38) | 9.47 (3.32) | 1.00 (.45) |
| **Parental self-efficacy**^o^ | 19.07 (4.28) | 18.94 (4.15) | 0.66 (.51) |
| **Psychological baseline stress**^i^ | 5.44 (2.97) | 5.33 (2.98) | 0.78 (.44) |
| **Parent-child dyad** |  |  |  |
| **Number of underaged children in household** | 1.68 (0.82; 1-13) | 1.66 (0.83; 1-13) | 0.47 (.64) |
| **Family’s place of residence** |  |  | 0.04 (.83) |
| Urban living | 659 (53.97) | 440 (53.40) |  |
| Rural living | 562 (46.03) | 384 (46.60) |  |
| **Family’s financial worries**^p^ |  |  | 2.00 (.16) |
| Yes | 237 (19.65) | 139 (17.06) |  |
| No | 969 (80.35) | 676 (82.94) |  |
| **Family’s first generation migration background**^q^ |  |  | 1.65 (.20) |
| Yes | 38 (3.18) | 36 (4.37) |  |
| No | 1158 (96.82) | 787 (95.63) |  |
| Abbreviations: N, absolute frequency; s.d., standard deviation.  ^a^ uncorrected for multiple comparisons; ^b^ no answer N=53 (baseline)/ N=32 (follow-up); ^c^ no, special school (*Förderschulabschluss*), or lower school certificate (Hauptschulabschluss); ^d^ secondary school certificate (Realschulabschluss) to university entry qualification (Abitur); ^e^ no answer N=1 (baseline)/ N=1 (follow-up); ^f^ in voluntary service, apprenticeship, national service, or jobseeking; ^g^ assessed with the Difficulties in Emotion Regulation Scale (DERS); ^h^ assessed with the Procrastination Questionnaire for Students (PFS, *Prokrastinationsfragebogen für Studierende*); ^i^ assessed with the Perceived Stress Scale (PSS-4); ^j^ no answer N=27 (baseline)/ N=1 (follow-up); ^k^ no or lower school-leaving certificate (*Hauptschulabschluss*); ^l^ secondary school-leaving certificate (*Realschulabschluss*) – doctor’s degree (PhD); ^m^ no answer N=1 (baseline)/ N=0 (follow-up); ^n^ no answer N=0 (baseline)/ N=2 (follow-up); ^o^ assessed with the Parenting Self-efficacy Questionnaire (FSW; *Fragebogen zur Selbstwirksamkeit in der Erziehung*); ^p^ no answer N=15 (baseline)/ N=9 (follow-up); ^p^ no answer N=25 (baseline)/ N=1 (follow-up). | | | |

| **Supplementary Table 2.**  Multilevel model on measurement time point with random effects (subjects and nesting parent-child dyads). | | | | | | |
| --- | --- | --- | --- | --- | --- | --- |
|  | **Coefficients** | **s.d.** | **t value** | **df** | **CI** | **P value** |
| **Fixed effects** | | | | | | |
| Intercept | 5.65 | 0.11 | 63.75 | 1160 | 5.47–5.82 | <.0001 |
| Lockdown | 1.15 | 0.11 | 13.66 | 1461 | 0.98–1.30 | <.0001 |
| **Random effects** | | | | | | |
| σ^2 a^ | 5.10 | 2.33 |  |  |  |  |
| τ_00_ _dyade:parent.child_ ^b^ | 1.18 | 1.11 |  |  |  |  |
| τ_00_ _dyade_ ^b^ | 2.60 | 1.61 |  |  |  |  |
| N _dyade_ | 731 |  |  |  |  |  |
| N _parent.child_ | 2 |  |  |  |  |  |
| ICC ^c^ | 0.43 |  |  |  |  |  |
| Observations | 2924 |  |  |  |  |  |
| Marginal R^2^ ^d^/conditional R^2 e^ | 0.035/0.446 |  |  |  |  |  |
| Abbreviations: CI, 95% confidence interval; df, degrees of freedom; s.d., standard deviation.  ^a^ sigma squared, population variance; ^b^ tau, between cluster residual; ^c^ Intraclass correlation coefficient; ^d^ proportion of variance associated with fixed effects; ^e^ proportion of variance associated with fixed and random effects. | | | | | | |

| **Supplementary Table 3.** Group comparisons: **adolescents** with and without stress increase. | | | | | |
| --- | --- | --- | --- | --- | --- |
|  | **No Stress Increase** | **Stress Increase** |  |  |  |
| **Variables** | **Mean (s.e.)/relative frequency in % [95%-CI]^e^** | **Mean (s.e.)/relative frequency in % [95%-CI]^e^** | **t/χ^2^/MANOVA post-hoc Scheffé value** | **P value** | **Cramér’s**  **V/ Cohen’s d** |
| **Absolute frequency** | 479 | 252 |  |  |  |
| **Relative frequency** | 65.53  [62.08-68.97] | 34.47  [31.03-37.92] |  |  |  |
| **Female sex** | 48.23  [43.75-52.7] | 46.43  [40.27-52.59] | 0.15 | .70 | 0.02 |
| **Age** | 13.08 (0.11) | 13.24 (0.16) | 0.83 | .40 | 0.07 |
| **First generation migration background** | 5.43  [3.4-7.46] | 2.79  [0.75-4.83] | 2.08 | .15 | 0.06 |
| **Number of underaged children in household** | 1.64 (0.03) | 1.65 (0.05) | 0.18 | .86 | 0.01 |
| **(Prospective) middle/high education level** | 91.34  [88.78-93.91] | 94.38  [91.52-97.24] | 1.71 | .19 | 0.04 |
| **High school student^a^** | 91.84  [89.39-94.29] | 94.05  [91.1-96.97] | 0.87 | .35 | 0.05 |
| **Financial worries** | 17.09  [13.7-20.48] | 18.33  [13.54-23.11] | 0.39 | .53 | 0.02 |
| **Urban living** | 47.18  [42.71-51.65] | 44.44  [38.31-50.58] | 0.10 | .75 | 0.03 |
| **Staying at home during CVLD** | 28.72  [24.66-32.78] | 23.81  [18.55-29.07] | 1.78 | .18 | 0.05 |
| **Emotion regulation^b^** |  |  |  |  |  |
| Limited access to emotion-regulation strategies | 6.39 (0.13) | 5.92 (0.16) | 0.47 | .03 | 0.17 |
| Non-acceptance of emotional responses | 6.49 (0.13) | 5.97 (0.16) | 0.52 | .02 | 0.19 |
| Impulse control difficulties | 5.91 (0.14) | 5.19 (0.18) | 0.73 | .002 | 0.24 |
| Difficulties engaging in goal-directed  behavior under unpleasant  emotions | 7.19 (0.14) | 6.79 (0.17) | 0.41 | .08 | 0.14 |
| Lack of emotional clarity | 6.53 (0.13) | 6.04 (0.17) | 0.48 | .03 | 0.18 |
| Emotional awareness | 8.24 (0.13) | 7.76 (0.16) | 0.47 | .03 | 0.17 |
| **Procrastination^c^** | 11.21 (0.18) | 10.72 (0.26) | 0.49 | .12 | 0.12 |
| **Parental stress baseline^d^** | 5.42 (0.14) | 5.16 (0.19) | 0.26 | .27 | 0.09 |
| **Parental stress during CVLD^d^** | 6.06 (0.13) | 6.89 (0.19) | -0.83 | <.001 | 0.29 |

Abbreviations: CVLD, Coronavirus lockdown; [95% CI], 95% confidence interval; s.e., standard error of means; MANOVA, Multivariate analysis of variance; post-hoc tests: t, t test, χ^2^ test, Scheffé test.

^a^ if “high school student” does not apply, subject is either in voluntary service, apprenticeship, national service, jobless, or jobseeking; ^b^ assessed with the Difficulties in Emotion Regulation Scale (DERS); ^c^ assessed with the Procrastination Questionnaire for Students (PFS, *Prokrastinationsfragebogen für Studierende*); ^d^ assessed with the Perceived Stress Scale (PSS-4).

| **Supplementary Table 4.** Group comparisons: **parents** with and without stress increase. | | | | | |
| --- | --- | --- | --- | --- | --- |
|  | **No Stress Increase** | **Stress Increase** |  |  |  |
| **Variables** | **Mean (s.e.)/relative frequency in % [95%-CI]** | **Mean (s.e.)/relative frequency in % [95%-CI]^e^** | **t/χ^2^/MANOVA post-hoc Scheffé value** | **P value** | **Cramér’s**  **V/ Cohen’s d** |
| **Absolute frequency** | 514 | 217 |  |  |  |
| **Relative frequency** | 70.31 [67-73.63] | 29.69 [26.37-33.00] |  |  |  |
| **Female sex** | 47.08 [42.77-51.4.] | 59.91 [53.39-66.43] | 9.54 | .002 | 0.12 |
| **Age** | 46.19 (0.36) | 47.16 (0.51) | 1.56 | .12 | 0.12 |
| **First generation migration background** | 4.47 [2.69-6.26] | 4.63 [1.83-7.43] | 0.00 | 1.00 | 0.00 |
| **Number of underaged children in household** | 1.67 (0.03) | 1.59 (0.05) | -1.43 | .16 | 0.11 |
| **Single parent** | 8.58 [6.15-11.00] | 3.24 [0.88-5.60] | 5.86 | .02 | 0.10 |
| **Middle/high education level** | 92.59 [90.33-94.86] | 89.4 [85.31-93.50] | 1.63 | .20 | 0.05 |
| **Full-/part- employment** | 89.49 [86.84-92.14] | 88.02 [83.7-92.34] | 0.21 | .65 | 0.02 |
| **Financial worries** | 17.91 [14.58-21.25] | 16.59 [11.64-21.54] | 0.10 | .75 | 0.02 |
| **Urban living** | 47.18 [42.71-51.65] | 44.44 [38.31-50.58] | 2.55 | .11 | 0.06 |
| **Staying at home during CVLD** | 49.22 [44.9-53.54] | 53 [46.35-59.64] | 0.72 | .40 | 0.03 |
| **Emotion regulation^a^** |  |  |  |  |  |
| Limited access to emotion-regulation strategies | 5.58 (0.12) | 4.95 (0.15) | 0.63 | .002 | 0.25 |
| Non-acceptance of emotional responses | 5.43 (0.11) | 5.38 (0.16) | 0.05 | .79 | 0.02 |
| Impulse control difficulties | 4.48 (0.1) | 4.1 (0.13) | 0.38 | .03 | 0.18 |
| Difficulties engaging in goal-directed  behavior under unpleasant  emotions | 6.49 (0.13) | 6.09 (0.16) | 0.40 | .07 | 0.15 |
| Lack of emotional clarity | 4.89 (0.10) | 4.4 (0.12) | 0.48 | .004 | 0.23 |
| Emotional awareness | 7.21 (0.11) | 6.69 (0.17) | 0.52 | .01 | 0.20 |
| **Procrastination^b^** | 9.63 (0.14) | 9.21 (0.23) | 0.41 | .12 | 0.13 |
| **Parental self-efficacy^c^** | 19.43 (0.16) | 19.96 (0.25) | -0.54 | .08 | 0.14 |
| **Childrens stress baseline^d^** | 5.98 (0.12) | 5.94 (0.21) | 0.04 | .85 | 0.30 |
| **Childrens stress during CVLD^d^** | 6.95 (0.13) | 7.89 (0.22) | -0.94 | <.001 | 0.58 |
| **Parental stress baseline^d^** | 6 (0.13) | 3.74 (0.15) | -11.13 | <.001 | 0.80 |
| **Parental stress during CVLD^d^** | 5.51 (0.12) | 8.33 (0.17) | 13.41 | <.001 | 1.06 |
| Abbreviations: CVLD, Coronavirus lockdown; [95% CI], 95% confidence interval; s.e., standard error; MANOVA, Multivariate analysis of variance; post-hoc tests: t, t test, χ^2^ test, Scheffé test.  ^a^ assessed with the Difficulties in Emotion Regulation Scale (DERS); ^b^ assessed with the Procrastination Questionnaire for Students (PFS, *Prokrastionationsfragebogen für Studierende*); ^c^ assessed with the Parenting Self-efficacy Questionnaire (FSW; *Fragebogen zur Selbstwirksamkeit in der Erziehung*); ^d^ assessed with the Perceived Stress Scale (PSS-4). | | | | | |

| **Supplementary Table 5.** Multivariate logistic regression model for psychological stress increase under coronavirus lockdown measures – **adolescent** full and reduced model using backwards elimination. | | | | | | | | |
| --- | --- | --- | --- | --- | --- | --- | --- | --- |
|  | **Initial full model** | | | | **Reduced model** | | | |
|  | **ß** | **SE** | **z value** | **P value** | **ß** | **SE** | **z value** | **P value** |
| **Intercept** | 0.12 | 0.82 | 0.14 | .89 | 1.14 | 0.28 | 4.13 | <.001 |
| **Urban living** | 0.08 | 0.19 | 0.44 | .66 | n.i. | | | |
| **Low (prospective) education** | -0.62 | 0.56 | -1.10 | .27 | n.i. | | | |
| **Female sex** | 0.10 | 0.19 | 0.51 | .61 | n.i. | | | |
| **Procrastination^a^** | 0.72 | 0.27 | 2.65 | .008 | 0.74 | 0.26 | 2.89 | .004 |
| **Non-acceptance of emotional responses^b^** | -0.05 | 0.28 | -0.16 | .87 |  |  |  |  |
| **Limited access to emotion regulation strategies^b^** | 0.67 | 0.32 | 2.12 | .03 | 0.70 | 0.26 | 2.69 | .007 |
| **Emotional awareness^b^** | -0.81 | 0.26 | -3.17 | .002 | -0.75 | 0.25 | -2.99 | .003 |
| **Family member’s stress increase^c^** | 0.86 | 0.21 | 4.12 | <.001 | 0.85 | 0.2 | 4.14 | <.001 |
| **Financial worries** | 0.78 | 0.26 | 2.99 | .003 | 0.76 | 0.26 | 2.96 | .003 |
| **Staying at home during CVLD** | 0.46 | 0.22 | 2.08 | .04 | 0.50 | 0.21 | 2.34 | .02 |
| **Migration background** | -0.38 | 0.55 | -0.69 | .49 | n.i. | | | |
| **High school student** | 0.46 | 0.40 | 1.16 | .24 | n.i. | | | |
| **Impulse control difficulties^b^** | -0.27 | 0.31 | -0.88 | .38 | n.i. | | | |
| **Difficulties engaging in goal-directed behavior under unpleasant emotions^b^** | 0.31 | 0.31 | 0.98 | .33 | n.i. | | | |
| **Lack of emotional clarity^b^** | 0.20 | 0.36 | 0.54 | .59 | n.i. | | | |
| **Significant covariates** | | | | | | | | |
| Age | 0.04 | 0.04 | 1.05 | .29 | n.i. | | | |
| Baseline stress^c^ | -0.48 | 0.05 | -10.25 | <.001 | -0.48 | 0.05 | -10.47 | 0.001 |
| Abbreviations: ß, standardized coefficient; CVLD, Coronavirus lockdown; SE, standard error; n.i., not included in final model after backwards elimination of predictors.  ^a^ assessed with the Procrastination Questionnaire for Students (PFS, *Prokrastionationsfragebogen für Studierende*); ^b^ assessed with the Difficulties in Emotion Regulation Scale (DERS); ^c^ assessed with the Perceived Stress Scale (PSS-4). | | | | | | | | |

| **Supplementary Table 6.** Multivariate logistic regression model for psychological stress increase under coronavirus lockdown measures – **parental** full and reduced model using backwards elimination. | | | | | | | | |
| --- | --- | --- | --- | --- | --- | --- | --- | --- |
|  | **Initial full model** | | | | **Reduced model** | | | |
|  | **ß** | **SE** | **z value** | **P value** | **ß** | **SE** | **z value** | **P value** |
| **Intercept** | -1.48 | 0.96 | -1.55 | .12 | -1.95 | 0.79 | -2.46 | .01 |
| **Urban living** | 0.34 | 0.19 | 1.80 | .07 | 0.37 | 0.19 | 1.96 | .05 |
| **Low (prospective) education** | 0.50 | 0.35 | 1.44 | .15 | 0.53 | 0.34 | 1.53 | .13 |
| **Female sex** | 0.63 | 0.20 | 3.20 | .001 | 0.64 | 0.19 | 3.30 | <.001 |
| **Procrastination^a^** | 0.54 | 0.28 | 1.95 | .05 | 0.53 | 0.27 | 1.95 | .05 |
| **Non-acceptance of emotional responses^b^** | 1.23 | 0.32 | 3.83 | <.001 | 1.11 | 0.27 | 4.09 | <.001 |
| **Limited access to emotion regulation strategies^b^** | 0.12 | 0.35 | 0.35 | .73 | n.i. | | | |
| **Emotional awareness^b^** | -0.15 | 0.26 | -0.58 | .56 | n.i. | | | |
| **Low parental self-efficacy^c^** | 0.47 | 0.30 | 1.55 | .12 | 0.44 | 0.30 | 1.49 | .14 |
| **Parental partnership** | 1.03 | 0.47 | 2.17 | .03 | 1.02 | 0.47 | 2.17 | .03 |
| **Family member’s stress increase^d^** | 0.46 | 0.20 | 2.37 | .02 | 0.47 | 0.19 | 2.40 | .02 |
| **Financial worries** | 0.59 | 0.28 | 2.11 | .03 | 0.62 | 0.28 | 2.25 | .02 |
| **Staying at home during CVLD** | 0.30 | 0.20 | 1.54 | .12 | 0.30 | 0.19 | 1.55 | .12 |
| **Part-time or full-time Occupation** | -0.14 | 0.31 | -0.45 | .65 | n.i. | | | |
| **Number of underaged children in household** | -0.10 | 0.14 | -0.75 | .46 | n.i. | | | |
| **Migration background** | 0.34 | 0.46 | 0.73 | .46 | n.i. | | | |
| **Impulse control difficulties^b^** | -0.08 | 0.34 | -0.23 | .82 | n.i. | | | |
| **Difficulties engaging in goal-directed behavior in the wake of unpleasant emotions^b^** | -0.07 | 0.39 | -0.18 | .85 | n.i. | | | |
| **Lack of emotional clarity^b^** | -0.39 | 0.32 | -1.22 | .22 | n.i. | | | |
| **Significant Covariates** | | | | | | | | |
| Age | 0.01 | 0.01 | 1.15 | .25 | 0.02 | 0.01 | 1.42 | .16 |
| Baseline stress^d^ | -0.43 | 0.05 | -9.16 | <.001 | -0.43 | 0.04 | -9.65 | <.001 |
| Abbreviations: ß, standardized coefficient; CVLD, Coronavirus lockdown; SE, standard error; n.i., not included in final model after backwards elimination of predictors.  ^a^ assessed with the Procrastination Questionnaire for Students (PFS, *Prokrastionationsfragebogen für Studierende*); ^b^ assessed with the Difficulties in Emotion Regulation Scale (DERS); ^c^ assessed with the Parenting Self-efficacy Questionnaire (FSW; *Fragebogen zur Selbstwirksamkeit in der Erziehung*); ^d^ assessed with the Perceived Stress Scale (PSS-4). | | | | | | | | |

## Recruitment

Households contacted for eligibility assessment (n=23 736)

exclusion criteria:

- non-respondents (N=11 309)
- no children in age of interest (10-17 years; N=10 694)
- did not provide necessary information or did not agree upon participation (N=512)

## Parent-child dyads taking part in baseline assessment (N=1221)

## Dyads taking part in follow-up (N=824)

excluded dyads (N=93)

- missing values of >25% in PSS-4

## Dyads considered for further analyses (N=731)

## Group 2

**No psychological stress increase**

Adolescents: N=479
Parents: N=514

## Group 1

**Psychological stress increase**

Adolescents: N=252
Parents: N=217

**Supplementary Figure 1.** Flow chart of sample recruitment and psychological stress groups
